# Supplementary material for: Emphasis on the deep or shallow parts of the tree provides a new characterization of phylogenetic distances
Source: Genome Biol. 2019 Jun 28;20:131. doi: 10.1186/s13059-019-1735-y (PMC6599322; doi:10.1186/s13059-019-1735-y)
Supplement: Supplementary file 1 — Supplemental methods and figures. Detailed description of simulations 1 and 2, Supplemental Figure S1, and Supplemental Figure S2. (PDF 78 kb) [file 13059_2019_1735_MOESM1_ESM.pdf]

Additional File 1: Supplemental Methods and Figures for *Deep or Shallow*

Julia Fukuyama

Simulations

In each of the simulations, we first create a matrix M representing the "true" abundance of each taxon in each sample, and we then create a matrix X, a noisy version of M, representing the "observed" abundance of each taxon in each sample. We then compute the branch contributions to the Unifrac distances in the observed taxon abundance matrix X and plot the branch contribution curves. The simulations differ in how the mean matrix M is created. The code used to perform the simulations and to generate the figures is available at https://github.com/jfukuyama/DeepOrShallow.

Simulation 1

In our first simulation, we have two groups of samples, each with its own set of characteristic taxa. The taxa in this simulation are related to each other by a randomly generated phylogenetic tree using the `rtree` function in `ape` [1], and the set of taxa characteristic of each group is unrelated to the phylogeny.

Let n denote the number of samples, p denote the number of taxa overall, n_{taxa} denote the number of taxa characteristic of each group. Our observed taxon abundance matrix $X \in \mathbb{R}^{n \times p}$ is

generated as

$$
\begin{aligned}
X_{ij} &\overset{\text{iid}}{\sim} \text{Poisson}(M_{ij}), \quad i = 1, \ldots, n, j = 1, \ldots, p \\
M &= \frac{10}{\|t\|} s {t^{(1)}}^T + \frac{10}{\|t\|}(1-s){t^{(2)}}^T + N \\
N_{ij} &\overset{\text{iid}}{\sim} \text{Gamma}(a, 1), \quad i = 1, \ldots, n, j = 1, \ldots, p \\
s &= \begin{pmatrix} \mathbf{1}_{n/2} \\ \mathbf{0}_{n/2} \end{pmatrix} \\
t^{(1)} &\sim \text{Uniform}\left(\left\{ x \in \{0,1\}^p : \sum_{i=1}^{p} x_i = n_{\text{taxa}} \right\}\right) \\
t^{(2)} &\sim \text{Uniform}\left(\left\{ x \in \{0,1\}^p : \sum_{i=1}^{p} x_i = n_{\text{taxa}} \right\}\right)
\end{aligned}
$$

The notation $\mathbf{1}_n$ represents the column vector containing all 1's of length n, and the notation $\mathbf{0}_n$ represents the column vector containing all 0's of length n. In the setup above, s denotes group membership, $t^{(1)}$ and $t^{(2)}$ are indicator vectors giving the taxa characteristic of the first and second groups, respectively, and a represents the mean abundance of the taxa that are not characteristic of either group.

Note that in this simulation strategy, for any pair (i, j) such that $s_i^{(1)} t_j^{(1)} + s_i^{(2)} t_j^{(2)} = 0$, the marginal distribution of X_{ij} is negative binomial, which is a standard distribution used to model counts in microbiome data [2].

We simulated taxon abundance matrices X using the strategy above for randomly selected values of n, p, n_{taxa}, and a. From the taxon abundance matrices and the corresponding phylogenetic tree, we computed normalized branch contributions to the Unifrac distances. The accumulation curves for these randomly selected values are given in Figure S1. We consistently see that unweighted Unifrac has a larger contribution from the shallow branches than weighted Unifrac, and that the generalized Unifrac distances have contributions somewhere in between. Interestingly, we see that at an absolute level, unweighted Unifrac places the most weight on the shallowest branches when the sparsity of X is low (most of the entries of X are non-zero) and places the least weight on the shallowest branches when the sparsity of X is high. In the high-sparsity case, we also see the most divergence between the weight placed on the deep vs. shallow branches between weighted and unweighted Unifrac. This finding is consistent with the mathematical result that the tree can be broken at branches that have descendants in all of the samples.

Simulation 2

In our second simulation, we have samples falling on a gradient, with the ends of the gradient corresponding to over- or under-representation of a certain clade. The taxa are again related to each other by a randomly generated phylogenetic tree.

Let n denote the number of samples, p denote the number of taxa overall, n_{taxa} denote the number of taxa in the clade corresponding to the gradient. Our observed taxon abundance

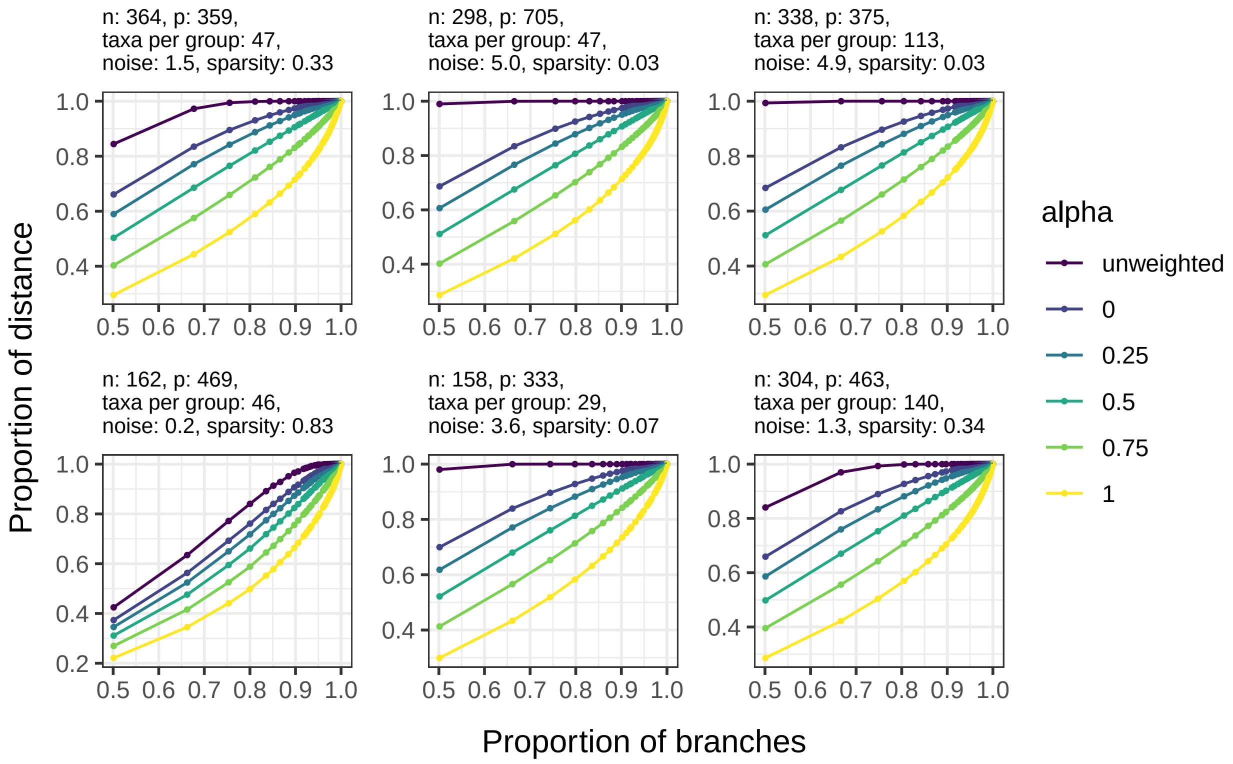

Figure S1: Cumulative average contribution (vertical axis) of the shallowest p fraction of the branches in the tree (horizontal axis) to unweighted and generalized Unifrac distances in simulation 1.

matrix $X \in \mathbb{R}^{n \times p}$ is generated as

$$X_{ij} \overset{\text{iid}}{\sim} \text{Poisson}(M_{ij}), \quad i = 1, \ldots, n, j = 1, \ldots, p$$
$$M = \frac{10}{\|t\|} st^T + N$$
$$N_{ij} \overset{\text{iid}}{\sim} \text{Gamma}(a, 1), \quad i = 1, \ldots, n, j = 1, \ldots, p$$
$$s_i \overset{\text{iid}}{\sim} \text{Uniform}(0, 1), i = 1, \ldots, n$$
$$c \sim \text{Uniform}(\{j : j \text{ is an interior node in the tree}\})$$
$$t = \begin{cases} 1 & \text{taxon } i \text{ descends from } c \\ 0 & \text{o.w.} \end{cases}$$

As before, s denotes group membership, t is an indicator vectors giving the taxa in the clade that varies along the gradient, and a represents the mean abundance for the taxa that are not involved in the gradient.

We simulated taxon abundance matrices X using the strategy above for randomly selected values of n, p, n_{taxa}, and a. From the taxon abundance matrices and the corresponding phylogenetic tree, we computed normalized branch contributions to the Unifrac distances and plotted the accumulation curves for these values. The results are shown in Figure S2. As before, we consistently see that unweighted Unifrac has a larger contribution from the shallow branches than weighted Unifrac, and that the generalized Unifrac distances have contributions somewhere in between.

References

[1] Paradis, E., Schliep, K.: ape 5.0: an environment for modern phylogenetics and evolutionary analyses in R. Bioinformatics (2018)

[2] McMurdie, P.J., Holmes, S.: Waste not, want not: why rarefying microbiome data is inadmissible. PLoS computational biology **10**(4), 1003531 (2014)

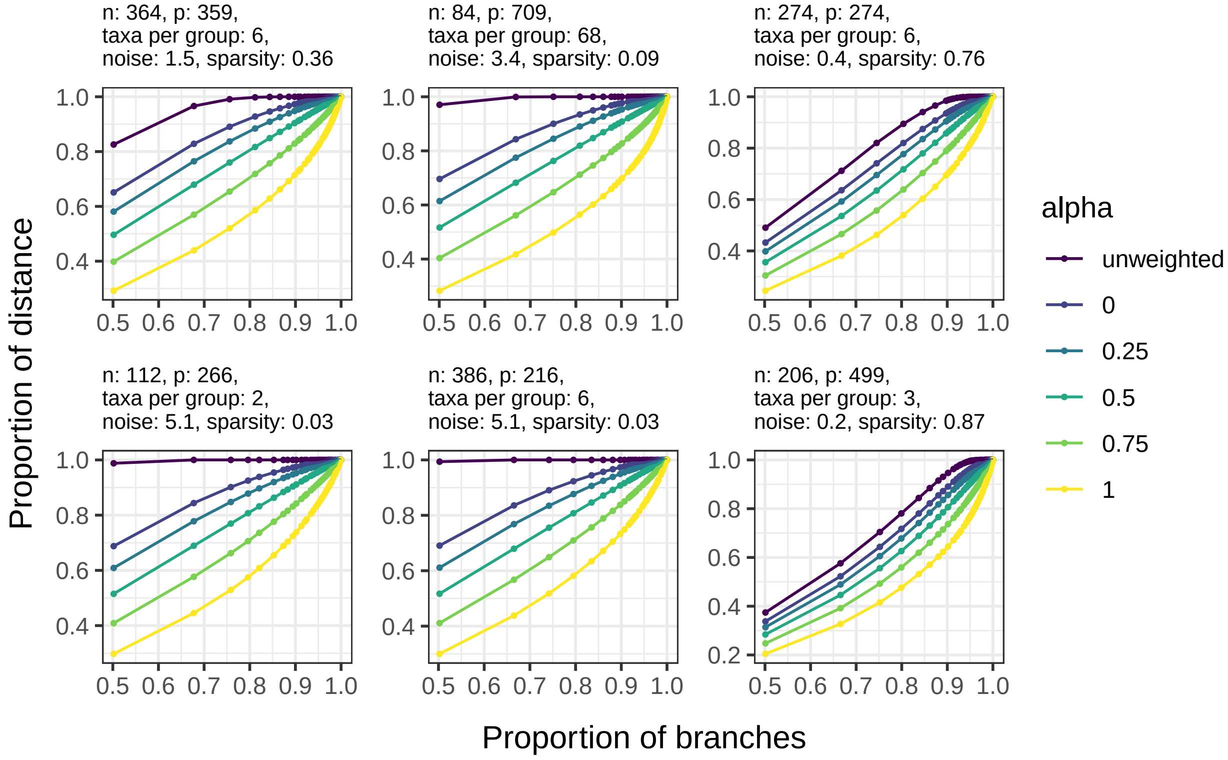

Figure S2: Cumulative average contribution (vertical axis) of the shallowest p fraction of the branches in the tree (horizontal axis) to unweighted and generalized Unifrac distances in simulation 2.
